# Decreased natural killer cell activity as a potential predictor of hypertensive incidence

**DOI:** 10.3389/fimmu.2024.1376421

**Published:** 2024-04-23

**Authors:** Yun-Kyong Lee, Eunkyung Suh, Hyoju Oh, Ji-Hee Haam, Young-Sang Kim

**Affiliations:** ^1^ Chaum Life Center, CHA University, Seoul, Republic of Korea; ^2^ Department of Family Medicine, CHA Bundang Medical Center, CHA University, Seongnam, Republic of Korea

**Keywords:** natural killer cells, natural killer cell activity, blood pressure, hypertension, incidence

## Abstract

**Introduction:**

Blood pressure is closely linked with immune function. This study examined the association between natural killer (NK) cell activity (NKA) and blood pressure and the development of hypertension according to NKA levels.

**Methods:**

This study enrolled 1543 adults who underwent NKA measurement and serial health check-ups at a medical center in Korea. NKA was estimated as the concentration of IFN-γ in the incubated whole blood containing a patented stimulatory cytokine. The participants were categorized into quartiles according to their NKA levels. Participants without hypertension were followed up, and the development of hypertension was compared according to the quartiles.

**Results:**

The prevalence of hypertension was not different among the NKA quartiles, whereas blood pressures significantly decreased, followed by an increment of quartiles (systolic blood pressure of 119.0 in Q1 and 117.0 in Q4, P-trend = 0.018). Over a mean follow-up period of 2.13 years, hypertension developed in 156 of 1170 individuals without baseline hypertension. The hazard ratio of Q4 compared with Q1 was 0.625 (95% CI: 0.397–0.983; p = 0.042).

**Conclusion:**

In conclusion, our findings indicate a correlation between lower NKA and higher blood pressure and the development of incident hypertension. This may suggest a potential protective role of NK cells against endothelial dysfunction. Further research is necessary to elucidate the specific relationship between immune functions and endothelial function.

## 1 Introduction

Hypertension is recognized as a risk factor for renal and cardiac diseases and stroke. Blood pressure is closely associated with endothelial inflammation and dysfunction, implying a connection between hypertension and immune function (1, 2). Evidence from animal models indicates that leukocyte subsets alterations may substantially contribute to the development of hypertension (3). Human studies have documented associations between the incidence and prevalence of hypertension and various cytokines and chemokines generated by leukocytes (4, 5).

Natural killer (NK) cells are a type of cytotoxic lymphocyte crucial to the innate immune system. They rapidly express many adhesion molecules common to the hematopoietic lineage, binding to the endothelium, extravasating and responding to chemotactic stimuli, similar to T cells (6). NK cells participate in vascular injury in hypertension (7). NK cell-derived interferon gamma (IFN-γ) plays a major role in activating monocytes/macrophages and driving them toward inflammatory phenotypes, which results in vascular dysfunction (8, 9). Findings from clinical studies are controversial pertaining to the association between circulating NK cells and endothelial function. In the MESA study, an increase in the number of NK cells was related to blood pressure (10). Conversely, patients with coronary heart disease showed a lower NK cytotoxic activity (11).

Established methods that measure NK cell activity (NKA), such as the ^51^Cr release assay and CD107a degranulation assay, have been widely employed to determine NK cell function. However, these methods are complicated and time-consuming as they require the isolation of peripheral blood mononuclear cells (PBMCs) or NK cells (12). To address these challenges, a relatively simple assay that employs whole blood instead of PBMCs or isolated NK cells was recently developed for commercial use to assess NKA. This innovative assay utilizes serum from *ex vivo*-stimulated whole blood to detect secreted IFN-γ from NK cells, serving as an NKA indicator (13). Clinical studies have indicated that NKA is a valuable marker for different cancers and is associated with immunological conditions such as ageing and vitamin D insufficiency (14–17).

The beneficial or harmful nature of NK cells in metabolic diseases remains a subject of debate (18). The depletion of NK cells reduces angiotensin II-induced vascular dysfunction, which is in contrast to their role in the inflammatory process (7). Currently, the role of NK cells in hypertension development has not been sufficiently evaluated. Specifically, the measurement of NKA has not been evaluated in this context. In this study, we examined the cross-sectional association between NKA, measured by IFN-γ released from activated NK cells, and blood pressure. Additionally, we examined differences in the incidence of hypertension according to baseline NKA levels over a mean period of 2.13 years.

## 2 Participants and methods

### 2.1 Study population

**Figure 1:**
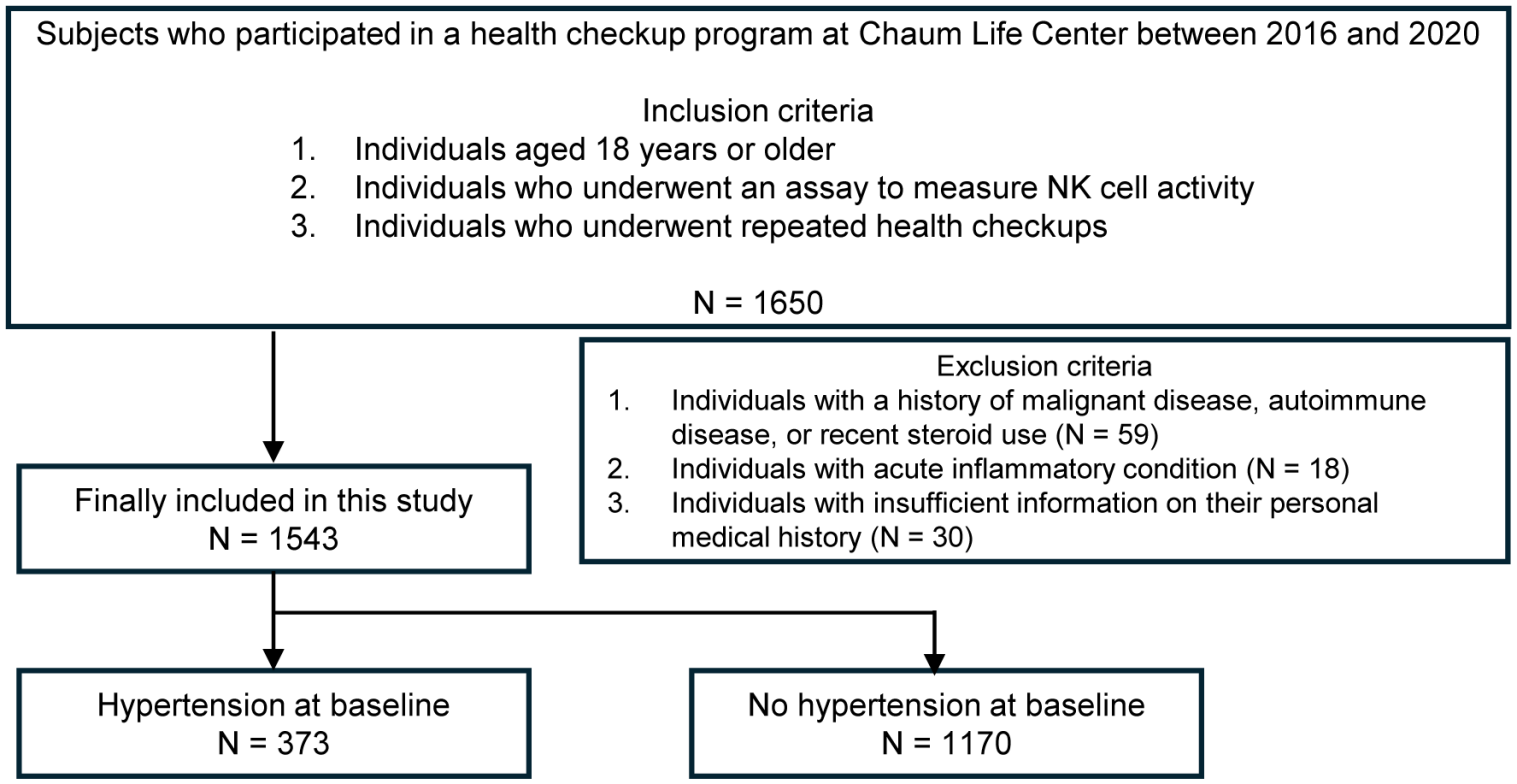
The study flow chart.

Health check-up data from the Chaum Life Center were analyzed in this study. We screened individuals aged 18 years or older who had participated in health check-up programs between 2016 and 2020. We included those who underwent an assay to measure NKA and attended repeated health check-ups (N = 1650). Among the eligible participants, we excluded individuals with a history of malignant diseases, autoimmune diseases or recent steroid use (N = 59) including those with acute inflammatory conditions (N = 18) and insufficient information on their personal medical history (N = 30), which were surveyed from all visits. Finally, a total of 1543 participants (768 men and 775 women) were included in the analysis (**Figure 1**).

The study protocol was approved by the Institutional Review Board of the CHA Bundang Medical Center (CHAMC 2020-10-006). Informed consent was waivered because of the retrospective nature of the study. This study was conducted in accordance with the principles of the Declaration of Helsinki.

### 2.2 Medical history of the participants

Medical histories and lifestyle habits of the participants were collected. The participants were classified into three categories according to their smoking habits: non-smokers, ex-smokers and current smokers. Significant alcohol consumption was defined as consuming >21 standard drinks/week in men and >14 standard drinks/week in women over a period of 2 years.

### 2.3 IFN-γ measurement for NKA

Using a 1-mL sample of whole blood, NKA was evaluated through measurement of IFN-γ, released by activated NK cells. This was performed by transferring whole blood directly into a tube containing a patented stimulatory cytokine (Promoca®, NKMAX, Seongnam, Korea), which causes secretion of IFN-γ into the plasma during the incubation period. The IFN-γ secretion predominantly occurs via the NK cells rather than through other innate or adaptive immune cells (13, 19–22). Within 30 min of collection, the tube was gently and repeatedly mixed and incubated for 20–24 h in a 37.0°C chamber. After incubation, the supernatant was obtained, centrifuged at 3000 × g for 3 min and then loaded onto enzyme-linked immunosorbent assay (ELISA) plates. The IFN-γ level was measured (pg/mL) using the designed ELISA.

### 2.4 Measurements

Height and weight were measured in a standing position without shoes and were recorded to the first decimal point in centimeters and kilograms, respectively. Body mass index (BMI) was defined as body weight in kilograms divided by height squared in meters. Blood pressure (BP) was measured using an automatic sphygmomanometer after resting for 10 min in a sitting position. The mean arterial pressure (MAP) was estimated using the following formula: (systolic BP + 2 * diastolic BP)/3.

### 2.5 Definition of hypertension

Hypertension was defined as meeting any of the following criteria: 1) Systolic BP ≥ 140 mmHg; 2) diastolic BP ≥ 90 mmHg; and 3) current use of antihypertensive medication (23).

### 2.6 Statistical analysis

Variables were summarized using mean and standard deviation or number and percentage as appropriate. Subsequently, the participants were stratified based on the presence of hypertension, and the variables were compared between the groups. The differences between the groups were compared using an independent sample t-test and a chi-square test.

To visualize the distribution of NKA, a histogram was drawn. The participants were further stratified into quartiles based on their IFN-γ levels and BP levels and the prevalence of hypertension were evaluated across these quartiles and compared using Pearson’s correlation test and Cochran–Armitage test for trend. To explore the association between risk factors and hypertension, logistic regression models were constructed.

For individuals without baseline hypertension, a longitudinal follow-up was conducted to observe the incidence of hypertension. Kaplan–Meier survival curves for each quartile were constructed and the Mantel–Haenszel test was used to compare hypertension development.

To elucidate the relationship between quartiles and the incidence of hypertension while adjusting for potential confounding factors, Cox regression models were developed.

All statistical analyses were conducted using R statistical packages version 4.3.3 (R Core Team, Vienna, Austria) and R studio 2023.06.0 + 421 with the gglot2 library. Statistical significance was determined at a conventional threshold of P < 0.05.

## 3 Results

Hypertensive participants were older, with a mean age of 54.2 years compared with 47.5 years in nonhypertensive participants (**Table 1**). The proportion of men was higher among hypertensive individuals. Furthermore, hypertensive individuals exhibited a higher BMI, with a mean of 25.3 compared to 22.7 in nonhypertensive individuals. While systolic BP, diastolic BP and MAP were higher in the hypertensive group, no difference was observed in the pulse rate between the two groups. Moreover, no significant difference was observed in the IFN-γ levels between the two groups.

**Table 1:** Baseline characteristics of the study participants.

	Total (N = 1543)	No hypertension (N = 1170)	Hypertension (N = 373)	P
Age (years)	49.1 ± 9.8	47.5 ± 9.4	54.2 ± 9.2	<0.001
Men (vs. women)	768 (49.8%)	501 (42.8%)	267 (71.6%)	<0.001
IFN-γ (pg/mL)	1120 ± 1002	1127 ± 1015	1099 ± 962	0.636
BMI (kg/m^2^)	23.3 ± 3.5	22.7 ± 3.4	25.3 ± 3.3	<0.001
Smoking				<0.001
Non-smoker	860 (55.7%)	720 (61.5%)	140 (37.5%)	
Ex-smoker	305 (19.8%)	221 (18.9%)	84 (22.5%)	
Current smoker	378 (24.5%)	229 (19.6%)	149 (39.9%)	
Alcohol consumer	1049 (68.0%)	768 (65.6%)	281 (75.3%)	0.001
Diabetes mellitus	58 (3.8%)	24 (2.1%)	34 (9.1%)	<0.001
Systolic BP (mmHg)	118.0 ± 13.9	113.6 ± 11.2	131.8 ± 12.4	<0.001
Diastolic BP (mmHg)	76.2 ± 11.4	72.5 ± 8.9	87.9 ± 10.5	<0.001
Pulse rate (bpm)	71.3 ± 9.9	71.2 ± 9.9	71.9 ± 9.8	0.234
Mean arterial pressure (mmHg)	90.2 ± 11.6	86.2 ± 9.0	102.5 ± 10.0	<0.001

The variables were compared between participants with and without hypertension. BP, blood pressure.

**Figure 2:**
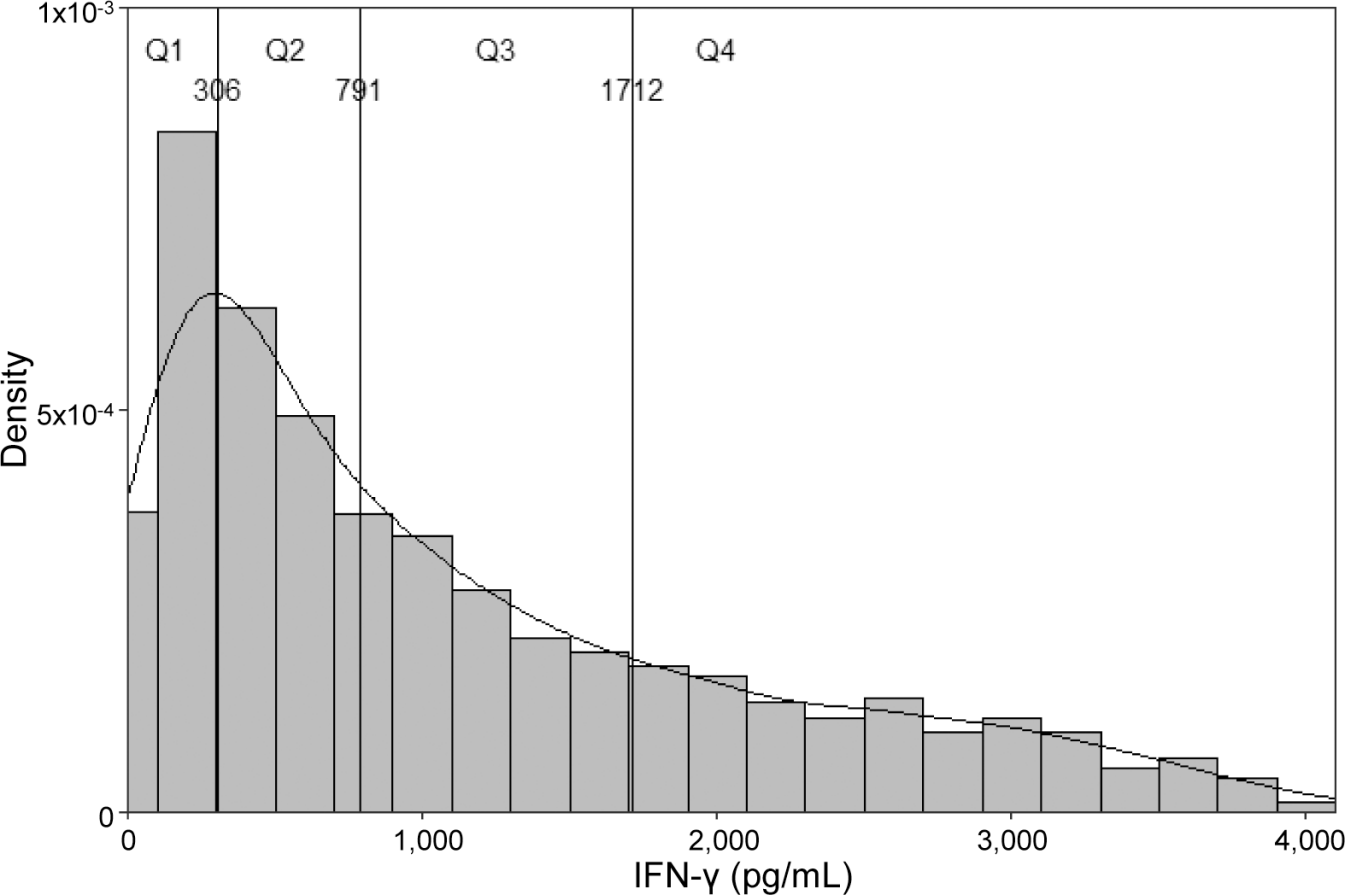
Distribution of IFN-γ and density plot of the study participants. Quartiles are drawn in the plot (Q1–Q4).

**Figure 3:**
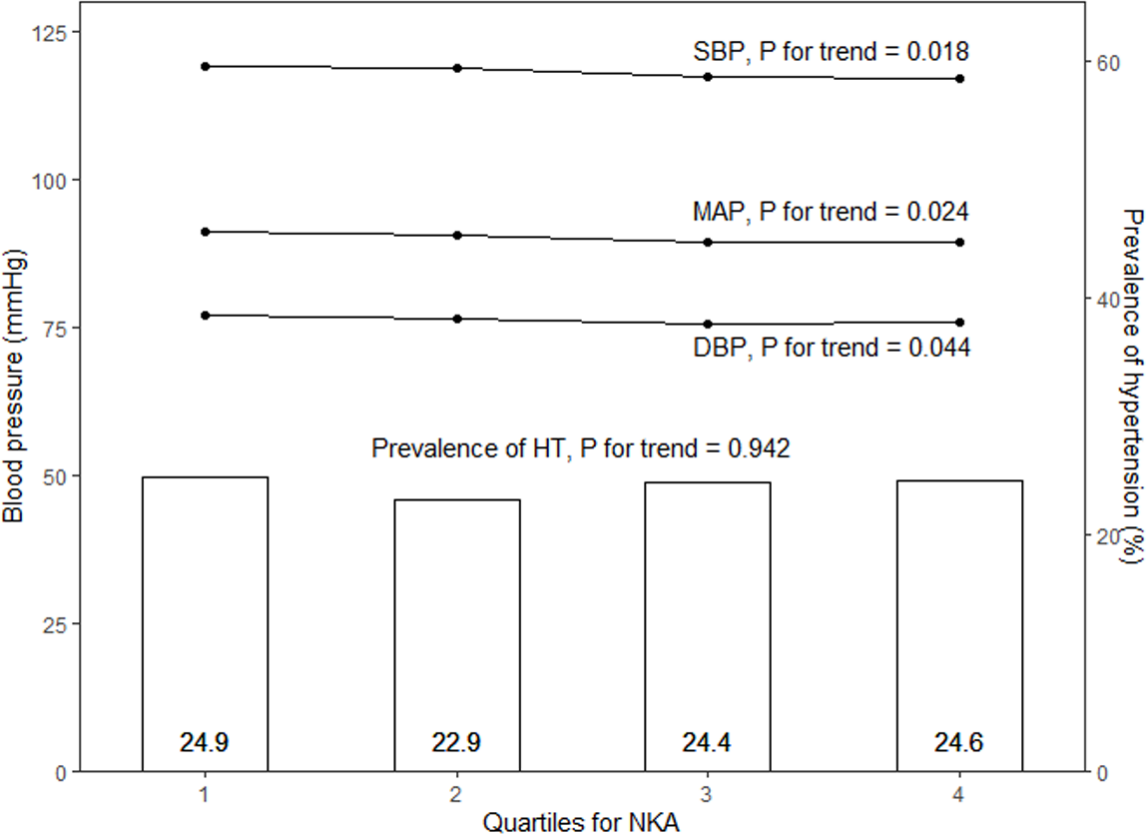
Blood pressure levels and the prevalence of hypertension are compared across the quartiles using Pearson’s correlation test and Cochran–Armitage test for trend. SBP, systolic blood pressure; MAP, mean arterial pressure; DBP, diastolic blood pressure; HT, hypertension; NKA, natural killer cell activity expressed in IFN-γ levels.

Based on the distribution of IFN-γ levels, the participants were divided into quartiles (**Figure 2**). According to this distribution, significant trends were observed in systolic BP, diastolic BP and MAP, all of which displayed meaningful differences across quartiles (**Figure 3**; p-values for trend were all statistically significant). In the multivariate logistic regression model (**Supplementary Table**), age, DM, BMI, and smoking emerged as significant predictors.

A total of 1170 individuals without baseline hypertension were included in the longitudinal analyses. Over a mean follow-up period of 2.13 years, hypertension developed in 156 individuals (13.3%). The incidence rate was calculated as 62.4 per 1000 person-years.

**Figure 4:**
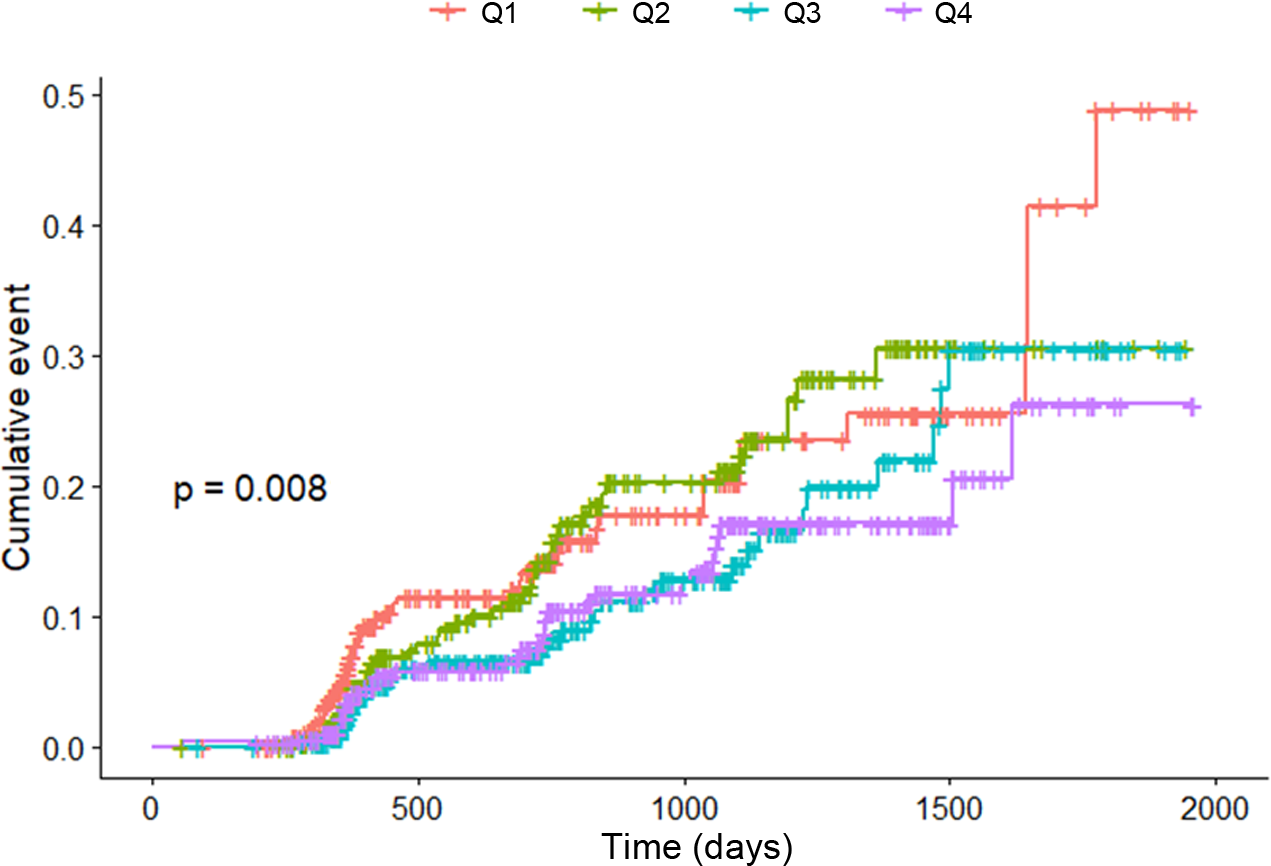
Kaplan–Meier curves for hypertension incidence according to quartiles.

Kaplan–Meier curves were employed in the analysis (**Figure 4**). The occurrence of hypertension was most frequent in the first quartile (Q1) and least frequent in the fourth quartile (Q4). The p-value for trend, calculated using the Mantel–Haenszel test, was determined to be 0.008.

**Table 2** shows the Cox proportional hazards model. In model 2, the HR for the third quartile (Q3) was 0.590 (95% CI: 0.376–0.926; p = 0.022) compared with Q1. Similarly, for the fourth quartile (Q4), HR was 0.625 (95% CI: 0.397–0.983; p = 0.042) compared with that for Q1. With each increase in the quartile, the HR was calculated as 0.836 (p = 0.011). In addition to NKA, age, sex, and BMI were also found to be significant factors (**Supplementary Table**).

**Table 2:** Cox regression models for hypertension incidence according to quartiles.

	Model 1		Model 2	
	Hazard ratio	P	Hazard ratio	P
Q1	Reference		Reference	
Q2	1.007 (0.663–1.529)	0.975	0.895 (0.587–1.366)	0.608
Q3	0.640 (0.409–1.003)	0.051	0.590 (0.376–0.926)	0.022
Q4	0.640 (0.407–1.006)	0.053	0.625 (0.397–0.983)	0.042
per quartile	0.836 (0.725–0.963)	0.013	0.829 (0.717–0.958)	0.011

Model 1 was adjusted for age and sex. Model 2 was additionally adjusted for body mass index, diabetes, alcohol consumption, and smoking.

## 4 Discussion

This study showed that a higher NKA was associated with a lower BP in a cross-sectional manner. Moreover, longitudinally, an increase in baseline NKA was linked to a reduced risk of developing hypertension. Specifically, individuals with an IFN-γ level below 300 pg/mL exhibited a 1.6-fold higher risk of incident hypertension than those with an IFN-γ level above 1700 pg/mL.

Conventional risk factors, including age, sex, obesity, metabolic disorders, dietary habits, physical activity, smoking, and alcohol consumption, are widely recognized as influential factors in blood pressure regulation and the development of hypertension (23, 24). In our study, several risk factors among these were found to be statistically significant. Unfortunately, data on dietary patterns and physical activity were not collected in our study. Literature indicates that diet and exercise may modulate immune function (25, 26). Previous research has examined the association between exercise and NKA, revealing disparities in NKA between women who engage in regular exercise and those who do not (17). Consequently, exercise may potentially influence the interplay between immune response and hypertension. Therefore, it can be suggested that the possibility of an exercise-immune interaction may have influenced our study findings.

In previous studies, the association between NK cells and BP has been controversial. NK cells have also been recognized as participants in the inflammatory process (27). In hypertension, NK cells are implicated in vascular injury and cytokines derived from NK cells impact vascular dysfunction (7). Notably, an increase in the number of peripheral NK cells has been linked to elevated BP (10). Conversely, NK cell dysfunction can render an individual more susceptible to atherogenic pathogens and potentially contribute to coronary artery disease (11). Consequently, a reduced NK cytotoxic activity is associated with coronary heart disease and atherosclerosis (28). These findings corroborate with the outcomes of our investigation.

Endothelial dysfunction may play a role in initiating and advancing vascular inflammation, vascular remodeling and atherosclerosis in hypertension, and it is independently linked to elevated cardiovascular risk (1). Various cytokines originating from NK cells could impact vascular inflammation and endothelial dysfunction (7). Conversely, NK cells might indirectly contribute to the preservation of endothelial function by regulating inflammation and angiogenesis (29–31). Our study provides support for the notion that NKA could play a role in attenuating the progression of hypertension.

Our study has several limitations. First, as it was conducted in a single center in Korea, the generalizability of our findings may be limited. However, it is important to note that our analysis involved a substantial number of participants and a comprehensive follow-up period. Subsequent investigations could help confirm the broader relevance of the correlation between NKA and hypertension. Second, our study did not use methods of quantifying NK cells. As recognized, the conventional method for assessing NKA is not suitable for accommodating a large sample size. Our employed method enabled the analysis of NKA in a substantial number of participants. The method used to measure IFN-γ levels as a surrogate marker for NKA also has limitation. IFN-γ concentrations may show slight variations depending on the specific duration of incubation within the range of 20–24 hours. However, minor deviations in incubation time are generally acceptable within clinical settings. Thirdly, several conventional risk factors such as diet and exercise were not included as covariates. Diet and physical factors likely influence the endothelium, potentially through mechanisms involving oxidative stress and vascular remodeling (32). Future studies should explore the interaction between diet/exercise and immune function.

Additionally, exploring how diet and exercise interact with the immune system to impact endothelial function warrants investigation.

In conclusion, our findings show a correlation between a lower NKA and higher BP, and the development of incident hypertension. Although our study found encouraging findings suggesting a potential protective role of NK cells against hypertension and endothelial dysfunction, further research is necessary to elucidate the specific relationship, particularly how NKA, and other immune functions, influence endothelial function. Further longitudinal studies could evaluate cardiovascular events according to NKA levels.

## Data availability statement

The raw data supporting the conclusions of this article will be made available by the authors, without undue reservation.

## Ethics statement

The studies involving humans were approved by Institutional Review Board of the CHA Bundang Medical Centre (CHAMC 2020-10-006). The studies were conducted in accordance with the local legislation and institutional requirements. The ethics committee/institutional review board waived the requirement of written informed consent for participation from the participants or the participants’ legal guardians/next of kin because We included the participants among the examinees with previous visit history with retrospective analysis design.

## Author contributions

YL: Data curation, Funding acquisition, Writing – original draft, Writing – review & editing. ES: Resources, Validation, Writing – original draft, Writing – review & editing. HO: Methodology, Software, Writing – original draft, Writing – review & editing. JH: Supervision, Validation, Writing – original draft, Writing – review & editing. YK: Conceptualization, Data curation, Formal Analysis, Funding acquisition, Investigation, Methodology, Project administration, Resources, Software, Supervision, Validation, Visualization, Writing – original draft, Writing – review & editing.
