# Supplementary material for: Decreased natural killer cell activity as a potential predictor of hypertensive incidence
Source: Front Immunol. 2024 Apr 23;15:1376421. doi: 10.3389/fimmu.2024.1376421 (PMC11074345; doi:10.3389/fimmu.2024.1376421)
Supplement: Supplementary file 1 [file Table_1.docx]

Supplementary table. Multivariate models for predicting hypertension prevalence and incidence

|  | OR (95%CI) | P |  | HR (95%CI) | P |
| --- | --- | --- | --- | --- | --- |
| NKA |  |  |  |  |  |
| Q1 | 1 |  |  | 1 |  |
| Q2 | 0.848 (0.584-1.230) | 0.386 |  | 0.895 (0.587-1.366) | 0.608 |
| Q3 | 1.011 (0.699-1.461) | 0.955 |  | 0.590 (0.376-0.926) | 0.022 |
| Q4 | 0.949 (0.657-1.372) | 0.781 |  | 0.625 (0.397-0.983) | 0.042 |
| Age (per 1y) | 1.077 (1.061-1.094) | <0.001 |  | 1.030 (1.014-1.046) | <0.001 |
| Women (vs. men) | 0.765 (0.529-1.108) | 0.156 |  | 0.495 (0.321-0.765) | 0.002 |
| Diabetes mellitus | 2.150 (1.201-3.898) | 0.010 |  | 0.616 (0.226-1.674) | 0.342 |
| Body mass index (per 1kg/m2) | 1.199 (1.149-1.253) | <0.001 |  | 1.129 (1.086-1.174) | <0.001 |
| Alcohol | 1.273 (0.920-1.769) | 0.147 |  | 0.912 (0.628-1.325) | 0.630 |
| Smoking |  |  |  |  |  |
| Non-smoker | 1 |  |  | 1 |  |
| Ex-smoker | 1.378 (0.919-2.062) | 0.120 |  | 1.488 (0.958-2.312) | 0.077 |
| Current smoker | 1.846 (1.285-2.658) | 0.001 |  | 0.878 (0.561-1.373) | 0.568 |
